# Supplementary material for: The Experience of Home Parenteral Therapy: A Thematic Analysis of Patient Interviews
Source: Pharmacy (Basel). 2023 Aug 22;11(5):133. doi: 10.3390/pharmacy11050133 (PMC10514802; doi:10.3390/pharmacy11050133)
Supplement: Supplementary file 1 [file pharmacy-11-00133-s001.zip › pharmacy-2540379-supplementary.pdf]

Supplementary 1 Patient questionnaire

Knowledge about home parenteral therapy or nutrition	Please tick your answer
Can you tell me the reason why you (the patient) were selected for home parenteral therapy or nutrition?	☐ Age<br>☐ Condition<br>☐ Type of therapy<br>☐ Patient wish<br>☐ Other:........
Do you think bed availability in the hospital could be one of the reasons a person can be initiated on a long-term home parenteral therapy or nutrition even if they are not well enough yet?	☐ Yes<br>☐ No<br>☐ Don't know
In your opinion, what is considered long enough and what is considered too long as a hospital stay, before initiating home parenteral therapy or nutrition?	Long enough: ....<br>Too long: ....<br>☐ Don't know
Who decides about the patient's suitability for home parenteral therapy or nutrition?	☐ Consultant<br>☐ SHO (Senior house officer)<br>☐ Nurse<br>☐ Other:........
What was the used assessment instrument for deciding about the patient's suitability for home parenteral therapy or nutrition?	☐ ....<br>☐ Don't know
Which guidelines were used?	☐ NICE guidelines<br>☐ Local Trust guidelines<br>☐ Don't know<br>☐ Other:........
How often is patients' suitability for home parenteral therapy or nutrition reviewed?	☐ 6-monthly<br>☐ Never<br>☐ When admitted to hospital for acute exacerbation<br>☐ Other:........
When you (the patient) were told about the home parenteral therapy or nutrition, who did advise you at the first instance?	☐ The health practitioner<br>☐ The nurse<br>☐ The specialist<br>☐ The SHO<br>☐ The pharmacist<br>☐ Other:........
After the initial advice, who provided you (the patient) with education and education materials?	☐ The health practitioner<br>☐ The ward nurses<br>☐ The specialist<br>☐ The SHO<br>☐ The pharmacist<br>☐ The district nurse<br>☐ Other:........
Did you (or the carer) receive any training in the aspects of home parenteral therapy or nutrition?	☐ Yes<br>☐ No
Do you think knowing the process is part of your initial training or you believe additional training is required?	☐ Yes<br>☐ No
What is your opinion about the training you received? Do you feel like you had enough training?	☐ The training has equipped you with all you needed to know<br>☐ You had to undertake self-directed training<br>☐ Additional training is required

Did the training include information about support services navigation and availability?	☐ Yes<br>☐ No
Did the training include information about infection control?	☐ Yes<br>☐ No
Did the training include information about medication handling and storage?	☐ Yes<br>☐ No
Did the training include information about consumables supply?	☐ Yes<br>☐ No<br>☐ N/A
Did the training include information about sharp disposals?	☐ Yes<br>☐ No<br>☐ N/A
Did the training include information about biologically contaminated consumables disposal?	☐ Yes<br>☐ No
Did the training include information about cytotoxic contaminated consumables disposal?	☐ Yes<br>☐ No<br>☐ N/A
In your opinion, what are the health challenges you faced as a result of parenteral home therapy?	☐ Social implications<br>☐ Ability implication<br>☐ Supply difficulty<br>☐ Health outcome implication<br>☐ Other:........
Who do you believe should also receive this type of training?	☐ Nurses<br>☐ Doctors<br>☐ Pharmacists<br>☐ Health workers<br>☐ Other:........
Who you believe should deliver this training to you?	☐ Nurses<br>☐ Doctors<br>☐ Pharmacists<br>☐ Health workers<br>☐ Other:........
How often you believe the health professionals' knowledge and techniques should be refreshed?	☐ Every 6 months<br>☐ Every year<br>☐ Every three years<br>☐ Other........
How often you believe health professionals should reassess the patients or their carers' knowledge and techniques?	☐ Every 6 months<br>☐ Every year<br>☐ Every three years<br>☐ Other........
Is there anything else I should have asked you or do you want to add?<br>If the answer is yes, then please write here:	

Supplementary 2 - HPT booklet - therapy specific content - IBD

Home Parenteral Therapy - IBD

Patient Support Information

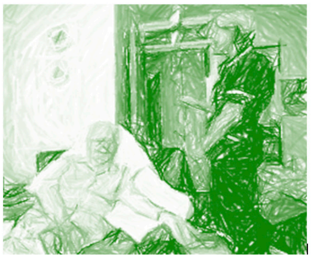

V4, 15/03/2020 – Mr Marko Puzovic (M.Puzovic@wlv.ac.uk), Dr Hana Morrissey (hana.morrissey@wlv.ac.uk) and Dr Patrick Ball (Patrick.ball@wlv.ac.uk)

Contents

Understanding the concept of Home Parenteral Therapy (HPT) 4
Clinical Home Healthcare 4
Home Healthcare Providers 5
Home Healthcare Provider Support 5
Your Responsibility 7
Change in Delivery Arrangement 8
Non-refrigerated Medicines and Medical Equipment 9
Refrigerated Medicine 10
Side effects 10
Clinical Waste and Sharps Bin Collection 11
Nursing Support 12
Data Protection and Confidentiality 13
Medical Information 13
Information the home healthcare provider will collect about you 13
Your information may also be used to help the home healthcare provider to: 14

Health Record 14
Understanding Your Medications 15
Infection Control 17
Hand Wash 17
Biological and Contaminated Waste 18
Signs of Infection and Red Flags 19

Understanding the concept of Home Parenteral Therapy (HPT)

Clinical Home Healthcare

The thought of receiving your treatment at home may be daunting for you. The purpose of this training is to explain how it all works and how you self-manage to improve your experience.

The clinical team at your hospital will prescribe your medicines according to your individual needs, and this will determine the frequency of deliveries to your home. The provider will call you to make arrangements for your first and subsequent deliveries of medicines and necessary medical supplies such as plasters and dressings.

When the providers contact you to arrange a delivery, they will:

- Confirm your delivery date
- Confirm the delivery address
- Check and record your current stock levels
- Advise you if your delivery driver will be collecting and replacing your sharps bin

The provider will provide a text reminder service the day before your delivery. The text message will advise you of your two-hour delivery window and the name of your delivery driver.

For some conditions, the provider might be able to loan you items such as a pump, drip-stand, refrigerator or dressing trolley depending on what your hospital or consultants says you need.

There are 3 basic types of intravenous access devices used for HPT: peripherally inserted central catheter (PICC) lines, midline catheters, and peripheral lines.

1.

Home Healthcare Providers

There are number of providers who support clinical home healthcare patients in the UK providing care to more than 36,000 patients in their own home – ranging from straightforward delivery of medication to specialist nursing for complex conditions.

Whether it's the provider drivers, the nurses, the patient services co-ordinators or the office support teams, they are all committed to one common purpose – supporting patients with their treatment at home.

Home Healthcare Provider Support

The support you will receive from your designated provider will be specific to you and your condition. Depending on what's been agreed with your clinical team at the hospital, it could be some or all of the following:

- Delivering medicine to you at home at regular intervals
- Providing all the necessary medical equipment for your treatment
- Nursing care support from highly qualified professionals
- Comprehensive training from a nurse where appropriate
- Clinical waste collection

For the purpose of this study parenteral therapy defined as: medication administered through the subcutaneous route or the intravenous route (directly or infused into the veins) through a previously established injection port.

We will use this sign whenever self-care is required:

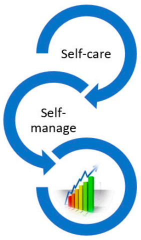

Self-care is defined as: A phrase used by the General Medical Council (GMC) (UK) referring to the duty that doctors have to encourage their patients to maintain good health through healthy lifestyle choices, and to educate patients and the public on how to maintain and improve their health.

Your Responsibility

Service providers take patient security and confidentiality very seriously. All of the drivers wear a uniform clearly indicating who they are, and they also carry photographic identification which they will be happy to produce upon request. Each delivery comes with a delivery note.

Make sure that you have received all the items that appear on this note. You should check that ***YOUR NAME*** appears correctly on all labels and check the medicine ***EXPIRY DATE***. When you are happy that the delivery is correct, sign the delivery note and hand it to the driver.

These two actions will ensure that you are receiving the medications prescribed for you and that they are safe to use.

It's important that you let your service provider know about any changes in your circumstances as soon as possible. Please let your home healthcare provider know if you:

- Move to a new house/change your address
- Change your telephone number
- Change your hospital or GP
- Want to change your delivery day/time

Change in Delivery Arrangement

Provided you inform your service provider at least ten working days in advance, they can arrange for your delivery to go to an alternative address. However, a named person (16 years or older) must be there to sign for the delivery on your behalf.

If you're going on holiday for a week or more or simply going to be away from home for a few days, you must let your provider know if this will affect your delivery arrangements. You need to inform the provider at least six weeks before you go away. This will help your provider to arrange a new delivery time and location to ensure that your service is not affected. You should always speak to your GP or consultant to make sure they are happy for you to travel.

- Sometimes circumstances mean you'll be away from home at short notice – even when this happens, please get in touch with your provider as soon as possible.
- If you are admitted to hospital, please ask them or a friend or member of your family to let the provider know as soon as possible.

The home healthcare provider needs to know so that they can cancel any deliveries or nurse visits that may have already been scheduled. Likewise, they will need to know when you will be returning home so that they can re-schedule your treatment.

2.

Non-refrigerated Medicines and Medical Equipment

Not all medicines are stored the same way so ***CHECK THE STORAGE INSTRUCTIONS*** on the dispensing label or the packaging.

It is important that your stock of medicines is used according to its expiry date. Each time you receive a delivery, make sure that the ***OLDEST STOCK IS USED FIRST***.

Medicines that do not need refrigeration should be stored in a cool, dry place away from direct sunlight or sources of heat such as radiators or fires.

Any equipment or ancillaries (e.g. gloves and dressings) that are provided by your service provider should be stored in a dry, cool place, away from heat and direct sunlight.

You need to notify your home healthcare provider if:

- You have a technical problem with any of your medication or equipment, for example injections. Do not dispose of any injections until you have spoken to them as they may need to return it to the manufacturer
- You feel unwell after taking medicine. You should also contact your GP or consultant.
- Your medicines are damaged in any way, or you believe they are unfit to use.
- You think any of the equipment or products that have been supplied are faulty. Provider may need to return the faulty product to the manufacturer, so please do not throw it away.

Refrigerated Medicine

Some medicines must be kept in a refrigerator. If this applies to you, the label will read

'Please store at 2–8°C'

You may be supplied with a fridge and a thermometer. Both are solely for medical use.

Please do not store food or drink in this fridge.

The fridge should be positioned where it is not subjected to extreme changes in temperature e.g. next to a fire or radiator, or in an outbuilding such as a shed or garage.

Allow it to reach the correct temperature before medicines are stored in it.

It is important to monitor the temperature inside the fridge daily and record the temperature.

Side effects

The side effects of your treatment are the same regardless of whether you have your treatment at home or in hospital, but if you have an adverse reaction, you or your nurse should report them immediately to your GP, the home healthcare provider or the hospital.

Clinical Waste and Sharps Bin Collection

You may be supplied with a yellow waste bin called a 'sharps bin'. This is for the safe disposal of clinical waste such as needles, glass vials and syringes.

Please do not use the bin for disposing of packaging material. The sharps bin should only be used for clinical waste (blood or bodily fluids, glass, needles another sharps) generated by the medicines or clinical supplies we deliver.

The provider should advise you when they will be collecting and replacing your sharps bin.

Please give the full sharps bin to the driver, ensuring that the lid is firmly closed and locked. If it is left open or is too full to close, the driver will be unable to take it away.

Nursing Support

Depending on your condition and the necessary treatment, your hospital or consultant may arrange for specialist nursing at home with your home healthcare provider. The home healthcare nurses are highly-qualified professionals and may perform a range of tasks for you including:

- Administering medication and changing dressing
- Educating and training you to administer your medication yourself
- Disconnecting/reconnecting you to your pump if you are a home parenteral nutrition (HPN) patient
- Discussing your treatment with you and explaining any side effect you experience
- Helping you understand how the treatment regime may affect you

All nursing staff providing home healthcare are registered with the Nursing and Midwifery Council and adhere to their Code. Your nursing care is provided in accordance with the procedures and instruction approved by the hospital that has referred you to the home healthcare provider.

The care plan is agreed on by the home healthcare provider, the NHS doctors and nurses and yourself.

Nursing support will be discussed with you beforehand if it is a necessary part of your treatment, and the number of visits you receive will be arranged on an individual basis. The home healthcare provider will contact you and confirm a two-hour time slot during which your nurse will arrive. Your nurse will also update your hospital or consultant so that they are aware of your progress.

3.

Data Protection and Confidentiality

The home healthcare provider is obliged to use appropriate security technology to safeguard your data and have procedures in place to ensure it remains confidential and is protected against unauthorised disclosure, use or loss. However, they may sometimes need to share your personal data with trusted third-party providers (e.g. specialised medical equipment supply) who will process your personal data on their behalf in accordance with the Data Protection Act.

Medical Information

Medical information will be kept confidential. It will only be disclosed to those involved with your treatment or care. Your home healthcare provider is required to send a summary of the care they have provided to your GP and/or consultant who referred you to them.

If you have not been referred by your GP and do not want any information to be sent to your GP, then please inform the nurse in charge of your care.

Information the home healthcare provider will collect about you

- Basic details about you, such as address and next of kin
- Contact history of deliveries, clinic visits, medicines administered
- Relevant information from other health professionals, relatives or carer(s)

Your information may also be used to help the home healthcare provider to:

- Review the care they provide to ensure it is of the highest standard
- Investigate adverse incidents or complaints
- Make sure their services meet the needs of our patients in the future
- Teach and train healthcare professionals
- Audit accounts and service
- Prepare statistics on performance

Health Record

Under the Data Protection Act, every living person (or their authorised representative) has the right to apply for access to their health records. It is not required to respond to requests for accessing health records unless it is provided with sufficient details to satisfy itself as to the identity of, or consent from, the individual making the request. There may also be a fee for the release of any information held.

All access requests should be sent in writing.

Understanding Your Medications

Home parenteral therapy for Inflammatory Bowel Disease (IBD) (Crohn's or Ulcerative Colitis)

Gut inflammation in Crohn's and Ulcerative Colitis is caused by over-activity of the immune system. Biologic medicines act to block some parts of this, reduce the inflammation and improve symptoms. These medicines may be an option when other drugs such as immunosuppressants (azathioprine, mercaptopurine, methotrexate) or steroids haven't been effective, or side effects have been hard to manage. There are a few different biologic medicines available to treat moderate to severe Crohn's Disease or Ulcerative Colitis but not all drugs are available to treat both conditions. All biologic medicines for self-administration at home are taken by injection under the skin and come ready to use in either a pre-filled syringe or a pre-filled injection 'pen'. You can't take biologics by mouth because the digestive system would break down and destroy the drug.

Drug	Adalimumab	Ustekinumab	Golimumab
**Brand Name**	Humira®, Amgevita®, Hulio®, Imraldi®, Hyrimoz®	Stelara®	Simponi®
**Used in**	Crohn's Disease<br>Ulcerative Colitis	Crohn's Disease	Ulcerative Colitis
**How it is taken**	Injection under the skin every 1 or 2 weeks	Injection under the skin every 8 or 12 weeks	Injection under the skin every 4 weeks

More information on biologic medicines can be found on Crohn's and Colitis UK website:
https://www.crohnsandcolitis.org.uk/about-crohns-and-colitis/publications/biologic-medicines

Adalimumab
https://www.crohnsandcolitis.org.uk/about-crohns-and-colitis/publications/adalimumab

Ustekinumab
https://www.crohnsandcolitis.org.uk/about-crohns-and-colitis/publications/ustekinumab

Golimumab
https://www.crohnsandcolitis.org.uk/about-crohns-and-colitis/publications/golimumab

One of the most common side effects of injections under the skin is pain at the injection site, sometimes with redness, itching and swelling. These tips can help:

- Allow your medication to warm to room temperature for 15-30 minutes before injecting
- Apply an ice pack, placed a light towel, before and after you inject to minimise pain
- Choose your injection site - regular injection site rotation allows proper absorption of the medication and decreases the risks of skin breakdown and scar tissue formation
- The most common sites used for the injection are the abdomen, thighs and upper arms
- Do not inject into areas where the skin is tender, bruised, red or hard
- Clean the skin with an alcohol wipe and leave to dry before injecting
- Use a good injection technique (see manufacturer's instructions in the Patient Information Leaflet)
- If you continue to have problems with pain at the injection site, you can ask your IBD team if you can try a different formulation of adalimumab

4.

Infection Control

Hand Wash

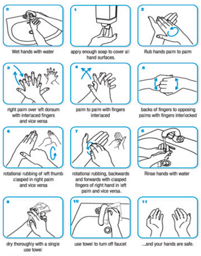

The World Health Organisation Hand Wash. Available at: http://www.who.int/gpsc/clean_hands_protection/en/

it is your responsibility to clean your hands before administering your treatment.

Biological and Contaminated Waste

Definition: waste that has been in contact with blood or other body secretions and must be discarded using specified procedures.

Contamination due to cytotoxic (chemotherapy) treatment are treated differently, including cloths and bed linen.

The guiding principle with biological waste is that all biological and contaminated material must be rendered harmless.

- **Yellow bags** marked with a black biohazard symbol should be used for clinical or infectious biological waste.
- **Red bags** marked with a radiation symbol should be used for radioactive waste, and
- **Purple bags** marked with the cell in telophase symbol should be used for cytotoxic waste.

You will need to ensure that your home healthcare provider has provided you with a workable arrangement for removing the biological and contaminated waste produced by the treatment you are receiving.

Signs of Infection and Red Flags

- Skin Spots
- Abnormal Heartbeat
- Urination Issues
- Agitation
- Impaired Focus
- Dizziness
- Accelerated Pulse
- Nausea & Vomiting
- Fever
- Chills
- Rapid Breathing
- Diarrhoea
- Pain and Weakness
- Pale and Cold Skin
- Low Blood Pressure

Make sure you report any ill feeling to your GP.

People livening with HIV or Hep C

Do not use other bins

Do not put used needles or other sharps in:

- *any type of household bin (for example, a general rubbish bin or a recycling bin)*
- *a container that's no longer needed, such as a drinks can or bottle*

Needles can cause injuries. Used needles can carry blood borne viruses that may be passed on to other people.

Viruses that can be passed on through contact with needles include:

- HIV
- hepatitis B
- hepatitis C
